# Tuberculous endocarditis: a case-based proposal for new diagnostic criteria

**DOI:** 10.5588/ijtldopen.25.0042

**Published:** 2025-08-13

**Authors:** M. Higa, H. Munakata, T. Yamazato, N. Abe, N. Ohyama, T. Fujii, J. Nambu, I. Nakazato, Y. Cho, M. Inamine, K. Takahashi, H. Katano, M. Narita

**Affiliations:** ^1^ Dept. of Respiratory Medicine, Okinawa Prefectural Nanbu Medical Center & Children’s Medical Center, Haebaru, Japan;; ^2^ Dept. of Respiratory Medicine, National Hospital Organization (NHO) Okinawa Hospital, Ginowan, Japan;; ^**3**^ Dept. of Cardiovascular Surgery, Okinawa Kyodo Hospital, Okinawa, Japan;; ^4^ Dept. of Cardiovascular Surgery, Okinawa Prefectural Nanbu Medical Center & Children’s Medical Center, Haebaru, Japan;; ^5^ Dept. of Diagnostic Pathology, Okinawa Prefectural Nanbu Medical Center & Children’s Medical Center, Haebaru, Japan;; ^6^ Dept. of Pediatric Infectious Diseases, Okinawa Prefectural Nanbu Medical Center & Children’s Medical Center, Haebaru, Japan;; ^7^ Dept. of Pathology, National Institute of Infectious Diseases, Tokyo, Japan;; ^8^ Division of Infectious Diseases, Dept. of Internal Medicine, Okinawa Prefectural Nanbu Medical Center & Children’s Medical Center, Haebaru, Japan;; ^9^ Division of Infectious Diseases, Dept. of Internal Medicine, Okinawa Prefectural Chubu Hospital, Uruma, Japan.

**Keywords:** TBE, Bentall procedure, gram staining of *Mycobacterium tuberculosis*, case report

## Abstract

**BACKGROUND:**

Tuberculous endocarditis (TBE) is a rare but often fatal manifestation of *Mycobacterium tuberculosis*. Although diagnosis is now possible with advanced techniques, the lack of standardized diagnostic criteria complicates timely recognition and management.

**METHODS:**

A 79-year-old man with a history of the Bentall procedure for annuloaortic ectasia, presented with fever and chest pain. Imaging revealed infective endocarditis with an aortic root abscess and vegetations. Histopathology identified granulation tissue with multinucleated giant cells, and *M. tuberculosis* was confirmed via PCR and culture. A literature review of TBE cases was performed to develop systematic diagnostic criteria.

**RESULTS:**

The diagnosis of TBE was established through histopathology and molecular methods. Based on this case and prior reports, diagnostic criteria for TBE were developed and categorized as ‘Definitive’, ‘Probable’, and ‘Possible’. These criteria incorporate clinical, microbiological, histological, and imaging findings to aid in diagnosis. The patient’s treatment included surgical intervention combined with antimicrobial therapy, aligning with strategies designed to improve outcomes.

**CONCLUSION:**

This case underscores the importance of considering TBE in infective endocarditis cases, especially those with atypical features. The proposed diagnostic criteria aim to improve the recognition and guide the management of TBE, emphasizing a multidisciplinary approach for better patient outcomes.

Tuberculous endocarditis (TBE) is a rare and diagnostically challenging condition. The first case of TBE was reported in 1859,^1^ and among all autopsy cases its prevalence ranges from 0.03–0.67%, including non-TB cases.^2,3^ Successful treatment of TBE, involving valve replacement and the administration of anti-TB drugs, was first reported in 1981,^4^ and advances in diagnostic techniques, particularly postoperative tissue culture, have led to an increase in TBE diagnoses.^5^ However, fewer than 50 cases of diagnosed TBE have been reported.^5,6^ TBE typically manifests as a disseminated *Mycobacterium tuberculosis* (*M. tuberculosis*) infection in immunocompromised and immunocompetent hosts.^7–17^ Here we report a case of TBE in an immunocompetent patient with an established diagnosis based on histopathological, microbiological and molecular biological findings.

## CASE PRESENTATION

A 79-year-old man presented with a 1-day history of fever and intermittent left-sided chest pain. He reported malaise and occasional low-grade fever for several months. At age 65, he underwent a Bentall procedure for annuloaortic ectasia, which involved replacement of the aortic valve with a bioprosthetic allograft. At age 75, he underwent a transurethral resection for bladder cancer, which had no recurrence. Notably, he had no history of TB or known TB exposure. He had a smoking history of 44 pack-years and had quit smoking at age 66. The patient’s vital signs were as follows: height, 161.0 cm; weight, 61.5 kg (body mass index: 23.7 kg/m^2^); body temperature, 36.9 °C; blood pressure, 99/68 mmHg; pulse rate, 93/min; respiratory rate, 20/min; and arterial oxygen saturation measured by pulse oximeter (SpO_2_), 95% on room air. Physical examination revealed an acutely ill general appearance with conjunctival petechiae. A maximal systolic ejection murmur was noted at the left sternal edge upon auscultation. No discernible Janeway lesions or Osler nodes were observed. Laboratory data indicated a creatine kinase (CK) level of 1,225 U/L, CK-MB level of 53.0 IU/L, troponin-I level of 0.62 ng/mL, and brain natriuretic peptide level of 281 ng/mL. The white blood cell count and C-reactive protein level were 6,210/mL and 1.43 mg/dL, respectively. Interferon-gamma release assays (IGRA: T-SPOT *TB*^@^) and HIV screening tests were both negative.

**Figure 1.**
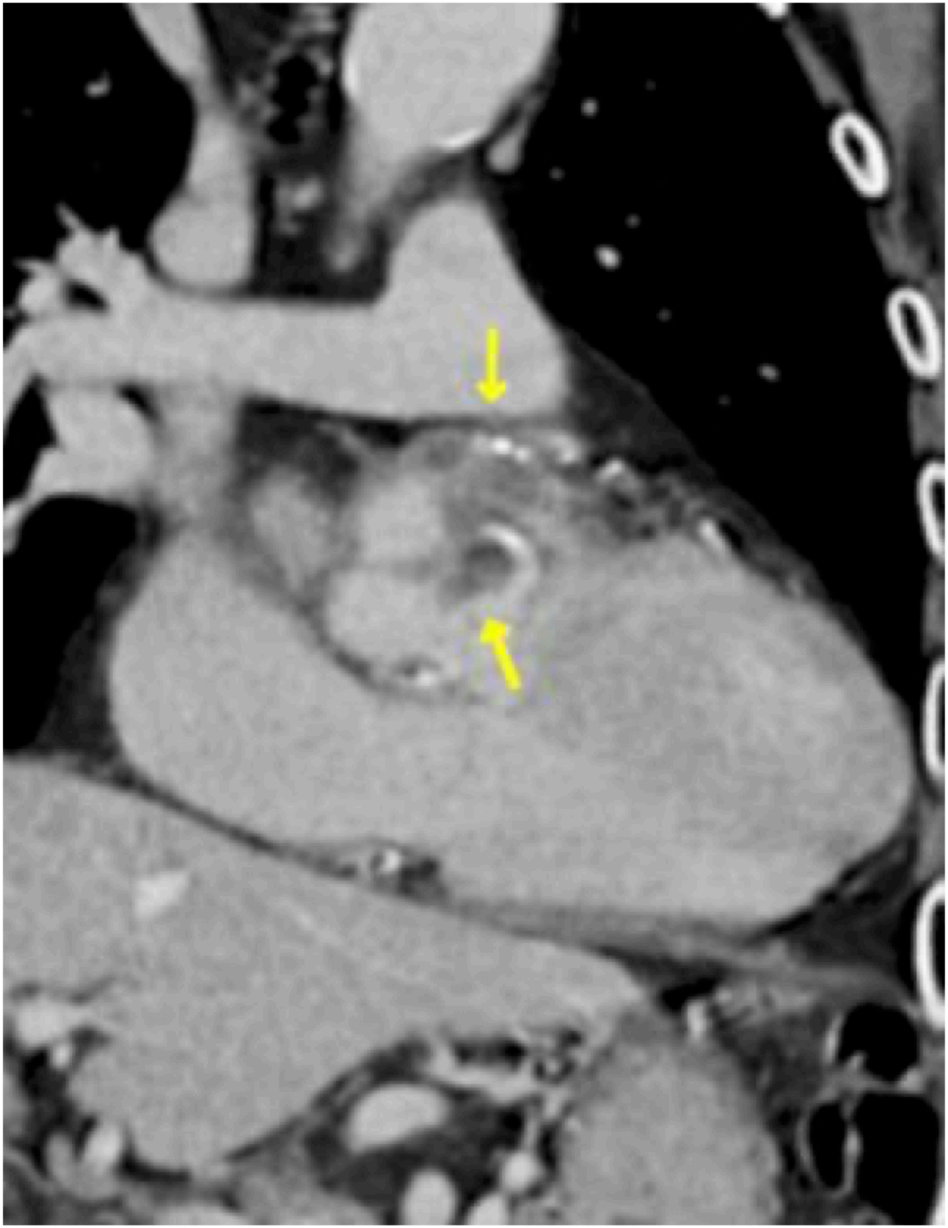
Chest computed tomography (CT) scan with contrast performed upon admission showing an area with poor contrast enhancement on the cranial side of the aortic root (yellow arrows), suggestive of an abscess.

**Figure 2.**
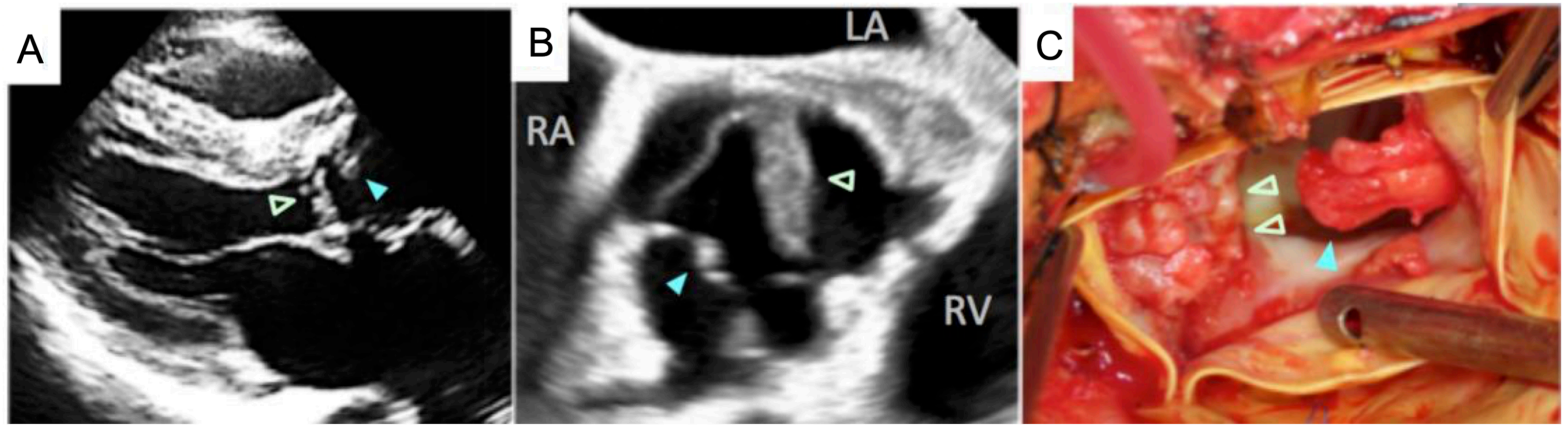
Aortic valve with vegetation (blue arrow: right coronary cusp; green arrows: left coronary cusps). **A**: Transthoracic echocardiography. **B**: Transesophageal echocardiography. **C**: Intraoperative findings. LA = left atrium; RA = right atrium; RV = right ventricle.

Chest radiography revealed cardiomegaly, while a chest computed tomography scan with contrast detected an area with poor contrast enhancement on the cranial side of the aortic root, suggestive of an abscess (Figure 1). Radiological investigation showed apical pleural thickening and microcalcification without filtration in both lungs. Echocardiography revealed aortic valve vegetation on the right and left coronary cusps of the aortic valve and an aortic perivalvular abscess (Figure 2). These echocardiographic findings indicated a Possible diagnosis of prosthetic valve endocarditis (PVE), prompting emergency valve replacement surgery on the first day of hospitalization.

**Figure 3.**
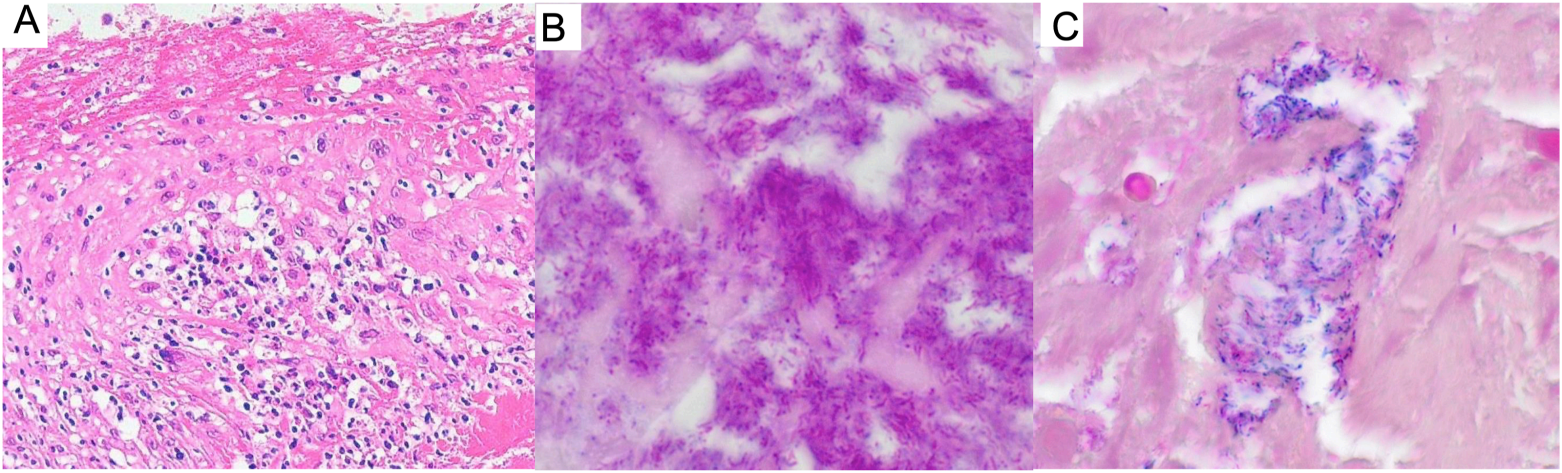
Histopathological findings of the vegetations. **A:** Hematoxylin and eosin staining revealing lymphocyte infiltration in the vegetation and non-necrotizing granulomas. **B:** Ziehl–Neelsen staining demonstrating positivity. **C:** Gram stain revealing numerous Gram-positive bacilli. Original magnifications: ×200 for A and ×400 for B and C.

Operative findings of the aortic valve revealed numerous vegetations on each cusp (Figure 2 panel C). Histopathological examination with hematoxylin and eosin staining of the vegetation revealed granulation tissue containing numerous plasma and multinucleated giant cells, cramped bacteria-like features, abscess formation, and necrosis. Additionally, acid-fast staining (AFS) of the tissue specimens was positive (Figure 3). Polymerase chain reaction (PCR) testing of a formalin-fixed paraffin-embedded tissue sample of the vegetation detected the presence of *M. tuberculosis.*^18^ A comprehensive search for pathogenic genes in the vegetation yielded negative results, except for *M. tuberculosis,*^18^ and *M. tuberculosis* was identified in the vegetation cultures. Mycobacterial testing of other specimens yielded negative results, including bronchial lavage fluid (collected during bronchoscopy to assess apical pleural thickening and microcalcification), as well as sputum, pleural effusion, blood, urine and stools. Gram staining of the aortic valve cusp revealed numerous bacilli, which appeared Gram positive (Figure 3), consistent with a significant mycobacterial presence. *Cutibacterium acnes* (*C. acnes)* was detected in one of four blood cultures (two out of eight bottles, both aerobic and anaerobic), but this was considered a contaminant.

Following a comprehensive evaluation of the clinical presentation, imaging findings, and laboratory results, a diagnosis of TBE was established. Hence, treatment was initiated with isoniazid (INH) at 5 mg/kg/day, rifampin (RFP) at 10 mg/kg/day, ethambutol (EB) at 15 mg/kg/day, and pyrazinamide (PZA) at 25 mg/kg/day. However, on treatment day 21, renal function declined, with creatinine clearance decreasing to less than 30 mL/min. This necessitated modifying the dosing schedule of EB and PZA to every other day. *M. tuberculosis* strains isolated from the vegetation culture were susceptible to INH, RFP, PZA and EB. Given the severity of TBE, a 12-month treatment regimen was implemented. This included 2 months of standard four-drug therapy, followed by 10 months of INH and RFP. No recurrence was observed during the 24-month follow-up period after completion of anti-TB treatment. In this case, despite the absence of a prior TB history or risk factors, bacteriological and histopathological findings confirmed the presence of *M. tuberculosis*, leading to the diagnosis of TBE.

This case report was approved by the Institutional Review Boards of Okinawa Prefectural Nanbu Medical Center and Children’s Medical Center (approval no. R6-102).

## DISCUSSION

TBE is an exceedingly rare condition with an unclear etiology. Historically, TBE has been frequently diagnosed during postmortem autopsies of patients with miliary TB. The first case of TBE was reported by Recklinghausen, who suggested that myocardial TB may progress from endocardial involvement, as observed in an autopsied cadaver with miliary TB.^1^ Recently, the rate of premortem diagnoses has increased.

Currently, no specific guidelines have been established for diagnosing TBE and *M. tuberculosis* is not recognized as a pathogen for infective endocarditis (IE) in the 2023 Duke-International Society for Cardiovascular Infectious Diseases Criteria for IE.^19^ In view of this, we propose the following diagnostic criteria, which are divided into three categories (Table 1): The ‘Definitive’ category is used when clear and strong evidence of TBE exists. A diagnosis is established if *M. tuberculosis* is identified through culture or PCR of a vegetation or abscess from the cardiovascular tissue. The ‘Probable’ category is considered when suggestive clinical and diagnostic findings are evident, but the presence of *M. tuberculosis* has not been confirmed through culture or PCR. This category requires that all of the following criteria be met: positive histopathological findings in the cardiovascular tissue sample (vegetation or abscess), positive acid-fast staining, positive echocardiographic findings (with or without additional imaging examinations), and evidence of *M. tuberculosis* infection, including positive blood culture test results. Non-tuberculous mycobacteria (NTM) are over 10 times more prevalent in patients with IE than *M. tuberculosis,*^20^ necessitating their differentiation. The ‘Possible’ category is used when the patient’s condition does not meet the criteria for ‘Probable’ TBE, as described above. Based on the criteria for 19 patients with TBE as outlined in Table 2, 32% (6/19) were classified as having ‘Definitive’ TBE,^7,8,11–13^ 11% (2/19) as having ‘Probable’ TBE^15,21^ and 58% (11/19) as having ‘Possible’ TBE^4,9,10,14,16,17,22–25^

**Table 1.** Criteria for diagnosing tuberculous endocarditis.

Definitive	*Mycobacterium tuberculosis* is identified via culture and/or polymerase chain reaction (PCR) of a vegetation or abscess from cardiovascular tissue. The tissue samples are typically obtained from native or prosthetic heart valves or an ascending aortic graft.
Probable	Suggestive clinical and diagnostic findings, but *M. tuberculosis* not confirmed by culture or PCR. Histopathology of cardiovascular tissue (vegetation or abscess) Caseous necrosis, granulomas, epithelioid cells, giant cells, or lymphocytic infiltration Positive acid-fast staining Imaging studies (echocardiography, computed tomography or magnetic resonance imaging) Abnormalities as vegetation, valvular/leaflet perforation, aneurysm/pseudoaneurysm, abscess, or fistula Evidence of *M. tuberculosis* infection, outside endocarditis (e.g., positive blood culture) This category requires that all the following criteria be met.
Possible	Does not meet criteria for Probable TBE as described above. Insufficient evidence to support a diagnosis of TBE renders the diagnosis less probable.

**Table 2.** Clinical characteristics of tuberculosis endocarditis over the past five decades.

Year	Age/sex	Comorbidities	US	Clues of TBE diagnoses	TBE criteria*	Anti-TB therapy	TD	Outcome	Ref
1981	20F	UNK	−	Vege AFS / H	Possible	RFP, INH, EB→INH, EB, Morinamide	UNK	ST	4
1984	60 F	UNK	UNK	Vege H, Sputum C	Possible	INH, EB, Steroids	UNK	Died	17
1984	76F	Af	−	Vege AFS / H, Lung tissue C	Possible	No treatment	None	Died	17
1990	78M	None	+	Liver, sputum, Urine C	Possible	RFP, INH, EB→RFP, INH, EB, PZA	UNK	ST	14
1996	56M	PTB	−	Vege AFS / H	Possible	RFP and INH	UNK	ST	16
1998	64M	CHF	−	Vege C / H	Definitive	INH, EB, PZA	12	ST	7
2002	35M	HIV, IVDU	+	Blood C	Possible	RFP, INH, EB, PZA→RFP, INH	UNK	ST	23
2007	63M	UNK	−	Vege H	Possible	RFP, INH, EB →RFP, INH	9	ST	22
2010	30M	None	+	Vege C	Definitive	RFP, INH, EB, PZA→RFP, INH	12	ST	8
2012	17F	None	+	Vege H	Possible	TB drugs (Details UNK)	UNK	ST	9
2014	61F	Steroid use	+	Vege AFS / H	Probable	No treatment	None	Died	21
2015	50M	None	−	Vege H, Sputum C	Possible	Four TB drugs (Details UNK)	9	ST	10
2015	21F	None	+	Vege AFS / H, Blood C	Probable	OFLX, INH, EB, PZA	12	ST	15
2016	1F	None	+	Vege PCR / AFS / H	Definitive	RFP, INH, EB, PZA	UNK	ST	11
2017	70F	UNK	+	Lymph node H	Possible	TB drugs (Details UNK)	9	ST	24
2018	37M	HIV, IVDU	+	Sputum AFS	Possible	RFP, INH, EB, PZA→RFP, INH	9	ST	25
2019	32M	BP	+	Vege C	Definitive	RFP, INH, SM, LVFX, DEX→INH, EB, MFLX, LZD →RFP, INH, EB, PZA MFLX, LZD	UNK	ST	12
2021	57M	None	+	Vege RT-PCR / H	Definitive	No treatment	None	Died	13
PC	79M	BP	+	Vege H / C / PCR /AFS	Definitive	RFP, INH, EB, PZA→RFP, INH	12	ST	None

M = Male; F = Female; Af: = Atrial fibrillation; TD = Total duration of treatment (months); UNK = Unknown; PTB = pulmonary TB; CHF = congestive heart failure; IVDU = ntravenous drug user; BP = Bentall procedure; US = ultrasound findings (vegetation/perivalvular or paravalvular abscess on ultrasound); TBE = tuberculous endocarditis; Vege = vegetation; AFS = acid fast staining; H = histology; C = culture; PCR = polymerase chain reaction; RT-PCR = real-time- polymerase chain reaction; RFP = rifampicin; INH = isoniazid; EB = ethambutol; PZA = pyrazinamide; OFLX = ofloxacin; SM = streptomycin; LVFX = levofloxacin; DEX = dexamethasone; MFLX = moxifloxacin, LZD = linezolid; ST = successful treatment; Ref = reference; PC = present case.

* TBE criteria defined in Table 1.

We compiled data from 19 patients with TBE, including those presented in Table 2, to accurately illustrate the clinical features of the condition.^4,7-17,21–25^ Since 1979, echocardiography has become the primary imaging modality for all suspected IE cases; therefore, we excluded TBE cases documented before 1979 in Table 2. Echocardiography has provided crucial diagnostic information in 63% (12/19) of TBE cases reported since 1980.^8,26^ The median age was 56 years (range, 1–79 years), and the male-to-female ratio was 11:8. Among the immunocompromised hosts, 11% (2/19) had HIV infection,^23,25^ 5% (1/19) had a history of steroid use (4 mg of methylprednisolone daily for 33 years)^21^ and the rest were immunocompetent^7–17^ or not described.^4,22,24^ The only pediatric patient, a 14-month-old infant, had a history of TB exposure.^11^ Additionally, 11% (2/19) had no history of TB exposure, whereas 84% (16/19) had an unknown TB exposure history. Among patients with TBE, 36% (7/19) had miliary TB^4,11,15,17,25^ whereas 47% (9/19) had pulmonary TB.^4,10-12,14,17,21,25^ Of note, 63% (12/19) had extrapulmonary TB, which involved the lymph nodes,^11,17,24,25^ liver,^14,15^ bone marrow,^11,15^ vertebrae,^13,17^ kidneys,^15^ esophagus,^17^ stomach,^17^ and pancreas.^17^ Only a minority of reported TBE cases (11%, 2/19) have shown positive results with IGRA^12,24^ and/or the purified protein derivative (PPD) skin test.^24^ However, 26% (5/19) showed no other TB-related findings, including exposure history, risk factors, complications, or examination results such as IGRA.^7-9,22^ Regarding surgical treatment, valve replacement for TBE was performed in 63% (12/19) of cases.^4,7-13,15,16,22^ Histopathological examination of the vegetation in 21% (4/19) of cases revealed evidence of TB, leading to a TBE diagnosis^7,9,22^ similar to the present case. Unlike Gram staining, AFS and vegetation culture are not routinely performed. Thirty-two percent (6/19) showed positive results for TB culture in the vegetation via PCR,^7,8,11–13^ 16% (3/19) showed positive results on acid-fast staining and histological examination, confirming TB in the vegetation,^4,15,16^ and the remaining 16% (3/19) were diagnosed based solely on the histological findings of the vegetation.^9,10,22^ Among those who did not undergo valve replacement for TBE treatment (37%, 7/19), two autopsied cadavers tested positive on AFS and/or had histological findings consistent with TB in the vegetation, leading to a TBE diagnosis.^17,21^ The remaining patients were diagnosed based on the results of liver tissue, sputum and urine cultures,^14^ blood cultures,^23^ sputum AFS^25^ and lymph node histology.^24^

All patients received anti-TB therapy, except for 16 % (3/19) who had fatal cases.^13,17,21^ Regarding treatment details, 16% (3/19) received a four-drug combination regimen (RFP, INH, PZA, and EB) for 2 months, followed by a two-drug therapy (RFP and INH).^8,23,25^ The treatment duration was 9–12 months in 42% (8/19).^7,8,10,15,22,24,25^ Among the cohort, 79% (15/19) recovered, 21% (4/19) died: two due to disseminated TB^17^ one because the family refused redo surgery after rebleeding,^13^ and the fourth (who had been using steroids for over three decades) due to acute respiratory distress syndrome accompanied by pulmonary TB.^21^

The first reported case of TBE occurring in a mechanical valve nine years after the Bentall procedure was documented in 2019.^12^ Our case, considered the second reported instance after the Bentall procedure, is unique as it occurred in a bioprosthetic valve allograft. PVE caused by *M. tuberculosis* is rare, with only a few cases reported.^27–29^ PVE typically occurs within the first postoperative year, often resulting from perioperative infection or contamination, and only rarely from the implantation of contaminated homografts.^27^ In contrast, late-onset PVE, which develops more than a year after surgery, is usually due to prosthesis seeding from transient bacteremia originating from a distant site, following the development of community-acquired infections caused by typical causative pathogens of IE.^30^ However, the mechanisms underlying the onset and progression of tuberculous PVE remain poorly understood, likely involving a complex interplay of host, pathogen, and environmental factors.

Blood culture-negative endocarditis (BCNE) is a form of IE in which standard blood culture methods cannot identify the causative microorganisms. In a prospective study of 819 patients with suspected BCNE, *M. tuberculosis* was identified in one of 49 valvular biopsy samples subjected to genetic testing using the primer extension enrichment reaction (PEER).^31^ The application of genetic testing methods, such as PCR or PEER, to cardiac valves or vegetation from patients with BCNE could facilitate the diagnosis of TBE, as demonstrated in this case report. Consequently, TBE should be considered in the differential diagnosis of BCNE. In this case, ***C. acnes*** was detected in one of the four blood cultures (two out of eight bottles, both aerobic and anaerobic). Although ***C. acnes*** is a well-established cause of both native and prosthetic valve endocarditis,^32^ a comprehensive genetic analysis of the valvular vegetation identified ***M. tuberculosis*** as the sole pathogen, with no detection of ***C. acnes***. Concurrent bacterial endocarditis due to ***C. acnes*** was therefore deemed unlikely and the final diagnosis was TBE. In the absence of other infectious foci, such as abscesses, the detection of *C. acnes* in blood cultures was considered as contamination.

The appearance of the valve was consistent with the presence of multiple Gram-positive bacilli. Although *M.tuberculosis* is considered Gram-variable and has been described as Gram-neutral (neither positive nor negative) or as a Gram-ghost^33–35^ (appearing unstained or as a faint, ghost-like shape on Gram staining) it may also appear Gram-positive due to the unique composition of its cell wall. Notably, the cell wall of *M. tuberculosis* exhibits features of both Gram-positive and Gram-negative bacteria.^36^ Furthermore, the process of pathological tissue fixation (using formaldehyde, alcohol, xylene, paraffin, thin sectioning, and subsequent treatment with xylene and alcohol) may have contributed to the Gram-positive appearance.

A limitation of this study lies in the diagnostic criteria proposed for TBE, which classify cases as ‘Definitive’, ‘Probable’, or ‘Possible’. The inclusion of ‘Possible’ TBE without direct pathological or molecular confirmation from valvular tissue, but based solely on supporting evidence such as suggestive findings of TB and echocardiographic findings,^14,23,24,25^ may increase diagnostic sensitivity but concurrently reduces specificity. This trade-off heightens the risk of overdiagnosis, particularly given the clinical overlap between mycobacterial endocarditis and other bacterial or non-infective etiologies. A more rigorous evaluation framework is needed to mitigate misclassification.

The diagnosis of TBE remains inherently challenging for several reasons. First, in the absence of overt risk factors for TB, AFS and mycobacterial culture of valvular tissue are not routinely performed resulting in potential underdiagnosis. Second, positive identification of non-tuberculous mycobacteria or fungi via MALDI-TOF mass spectrometry may reflect contamination rather than true infection, complicating the interpretation of microbiological findings. Third, reliance on PCR for *M. tuberculosis* detection has inherent limitations, including susceptibility to false-positive and false-negative results, and thus cannot alone support a Definitive diagnosis. Furthermore, confirmation of TBE through clinical response to anti-TB therapy is not feasible once the affected valve has been surgically excised. The complexity of patient comorbidities (such as immunosuppression e.g., HIV infection, diabetes) and social risk factors (e.g., homelessness,^37^ or intravenous drug use^38^) further obscures the diagnostic picture. These conditions may predispose patients to multiple overlapping pathologies, including concurrent pulmonary TB and bacterial endocarditis, confounding the determination of the true etiology of valvular vegetations. To enhance diagnostic precision and avoid misclassification, a comprehensive approach integrating histopathological examination, microbiological culture and molecular diagnostics is essential.

## CONCLUSION

This review focuses on a case series, which includes the case described here, and emphasizes the clinical importance of considering TBE as a potential cause of prosthetic valve infection and unexplained endocarditis, even in the absence of traditional TB risk factors. TB is a rare cause of endocarditis, and although it is infrequently considered in clinical practice, there is a significant risk of misdiagnosing other causes of endocarditis as TBE due to the limitations of current TB diagnostics and the challenges in accessing the infected tissue. Although data to guide management are extremely limited, early treatment is likely to be critical to survival. Therefore, this rare diagnosis should be considered in cases that meet the proposed diagnostic criteria.
